# CD16xCD33 Bispecific Killer Cell Engager (BiKE) as potential immunotherapeutic in pediatric patients with AML and biphenotypic ALL

**DOI:** 10.1007/s00262-021-03008-0

**Published:** 2021-08-16

**Authors:** Sarah B. Reusing, Dan A. Vallera, Angela R. Manser, Titus Vatrin, Sanil Bhatia, Martin Felices, Jeffrey S. Miller, Markus Uhrberg, Florian Babor

**Affiliations:** 1 Institute for Transplantation Diagnostics and Cell Therapeutics, Heinrich Heine University, Düsseldorf, Germany; 2 Department of Pediatric Oncology, Hematology and Clinical Immunology, Centre for Child and Adolescent Health, Medical Faculty, Heinrich Heine University, Moorenstraße 5, 40225 Düsseldorf, Germany; 3 Department of Therapeutic Radiology-Radiation Oncology, Masonic Cancer Center, University of Minnesota, Minneapolis, MN USA; 4 Department of Medicine, Division of Hematology, Oncology and Transplantation, Minneapolis, MN USA

**Keywords:** Biphenotypic ALL, NK cells, Antibody therapy, Bispecific antibodies, CD33, BiKE

## Abstract

Similar to pediatric acute myeloid leukemia (AML) the subgroup of biphenotypic acute lymphoblastic leukemia (ALL) is a rare complex entity with adverse outcome, characterized by the surface expression of CD33. Despite novel and promising anti-CD19 targeted immunotherapies such as chimeric antigen receptor T cells and bispecific anti-CD19/CD3 antibodies, relapse and resistance remain a major challenge in about 30% to 60% of patients. To investigate the potential role of the fully humanized bispecific antibody CD16 × CD33 (BiKE) in children with CD33^+^ acute leukemia, we tested whether the reagent was able to boost NK cell effector functions against CD33^+^ AML and biphenotypic ALL blasts. Stimulation of primary NK cells from healthy volunteers with 16 × 33 BiKE led to increased cytotoxicity, degranulation and cytokine production against CD33^+^ cell lines. Moreover, BiKE treatment significantly increased degranulation, IFN-γ and TNF-α production against primary ALL and AML targets. Importantly, also NK cells from leukemic patients profited from restoration of effector functions by BiKE treatment, albeit to a lesser extent than NK cells from healthy donors. In particular, those patients with low perforin and granzyme expression showed compromised cytotoxic function even in the presence of BiKE. In patients with intrinsic NK cell deficiency, combination therapy of CD16xCD33 BiKE and allogeneic NK cells might thus be a promising therapeutic approach. Taken together, CD16xCD33 BiKE successfully increased NK cell effector functions against pediatric AML and biphenotypic ALL blasts and constitutes a promising new option for supporting maintenance therapy or “bridging” consolidation chemotherapy before hematopoietic stem cell transplantation.

**Supplementary Information:**

The online version contains supplementary material available at 10.1007/s00262-021-03008-0.

## Introduction

Acute leukemia is the most common malignancy diagnosed in children and represents approximately 30% of pediatric cancer diagnoses [1]. The vast majority (80%) suffers from acute lymphoblastic leukemia (ALL) while the remainder is diagnosed with acute myeloid leukemia (AML). Among these, approximately 25% of biphenotypic, or bilineage ALL express the myeloid specific antigen CD33. While patients with common B-precursor ALL have excellent prognosis, acute lymphoblastic leukemias with.

Co-expression of CD33 usually have a poor prognosis [2, 3]. Thus, patients with CD33^+^ leukemia mark a high-risk population urgently requiring novel strategies that promote the immune system to overcome the malignancy without adding further life-threatening toxicity.

Through their ability to control human hematologic malignancies and to exhibit antitumoral effects, natural killer (NK) cells represent key players of the innate immune system, capable of immune surveillance [4]. The modulation of NK cell activity is regulated by a repertoire of activating and inhibitory receptors. Ultimately, the balance of these receptors will determine whether an NK cell will be silent (tolerant), auto-reactive, or cytotoxic (alloreactive) toward the healthy or malignant “self.” NK cell function can be accomplished via I) natural cytotoxicity against tumor target cells upon degranulation of lysosomes containing granzymes and perforin. II) Cytokines such as interferon γ (IFN-γ) and tumor necrosis factor α (TNF-α) help shaping the adaptive immune response and III) via CD16, the potent low-affinity FcγRIII receptor, mediating antibody-dependent cell-mediated cytotoxicity (ADCC) [5]. The vast majority (> 90%) of circulating NK cells are CD56^dim^ and express high levels of CD16 [6]. CD16 induces phosphorylation of immunoreceptor tyrosine-based activation motifs (ITAM), triggering the release of lytic granules such as granzyme and perforin and cytokines such as INF-y and TNF-α [7, 8]. Different studies have demonstrated the therapeutic potential of manipulating NK cells via CD16 and monoclonal therapeutic antibodies [9, 10]. In this context, bispecific antibodies represent a novel class of monoclonal antibodies that link surface antigens on tumor cells to effector cell receptors of cytotoxic lymphocytes such as NK cells, thereby creating an antineoplastic effect. These antibodies are characterized by specificity against a target expressed by the malignant cell population or playing a critical role for neoplastic cell development. To more efficiently direct NK cells to leukemic targets, a fully humanized bispecific Killer Cell Engager has been designed recently [11, 12]. The CD16xCD33 BiKE comprises two antibody fragments, a first recognizing CD16 (FcyRIII) and a second, directed against the myeloid differentiation antigen CD33, which together trigger antibody-dependent cell-mediated cytotoxicity [11, 12]. Engagement of CD16 signaling against CD33^+^ targets is NK cell specific and targets CD33^+^ cells exclusively. Thus, the antibody directly triggers NK cell activation through CD16, significantly increasing NK cell cytotoxicity and cytokine production. Moreover, by this means CD16xCD33 BiKE has been shown to potentially overcome the inhibitory effect of KIR signaling and improve NK cell-mediated lysis of AML blasts derived from adults. In the present study, we evaluated whether CD16xCD33 BiKE could enhance NK cell activation against CD33^+^ primary childhood ALL and AML cells.

## Material and methods

### Patient samples

Primary CD33^+^ blasts from pediatric patients with ALL and AML were obtained from patients from the Department of Pediatric Oncology, Hematology and Immunology of the University Clinic, Düsseldorf (Director Prof. A. Borkhardt). CD33 expression levels in peripheral blood ranged from 30 to 70% in the ALL and AML patients.

### Cell lines

The three human cell lines SEM (CD33^±^), HL60 (CD33^+^) and Raji (CD33^−^) were used as positive and negative controls for CD33 expression.

### Cell isolation and purification

Peripheral blood mononuclear cells (PBMC) were isolated from patients, healthy donors and buffy coats (Blood Donation Center Düsseldorf, Germany) using density gradient centrifugation with Biocoll Separating Solution (Biochrom, Berlin, Germany). For PBMC purification, CD33^+^ monocytes and granulocytes were depleted using a magnetic-activated cell sorting (MACS) CD33 Isolation Kit protocol (Miltenyi Biotec).

### Immunofluorescence and flow cytometry

The following fluorescence-labeled monoclonal antibodies were used: CD3 (UCHT1), CD14 (HCD14), CD16 (3G8), CD33 (P67.6), CD45 (HI30), CD56 (HCD56), granzyme B (GB11), perforin (dG9), CD107 (H4A3), interferon-γ (B27) and tumor necrosis factor-α (MAb11), all purchased from Biolegend (CA, USA).

### CD107a mobilization assay, cytokine production

CD33 depleted PBMC were cultured overnight in RPMI 1640 containing 10% fetal bovine serum, 5% human serum type AB (Biochrom) and 1000U/mL interleukin-2 (Novartis, Basel, Switzerland). Cells were harvested and treated with or w/o 10 µg/mL CD16xCD33 for 30 min prior to incubation. HL60 leukemic targets and CD33-depleted PBMCs were added and cocultured with (E:T) ratio of 10:1 in a volume of 200μL in a 96-well plate. After incubation for 1 h, 2μL of 2 mM Monensin (Biolegend) and 10 µg/mL of Brefeldin (Sigma-Aldrich, Missouri, USA) were added and incubated for a further 5 h. CD107 expression and intracellular IFN-ɣ and TNF-α were evaluated as previously described.

### Cytotoxicity assay

HL60 target cells were stained with CFDA-SE (Vybrant®CFDA-SE Tracer Kit, Invitrogen, CA, USA). PBMC were cultured overnight with interleukin-2 1000U/mL. Stained target cells and effector cells were mixed at a ratio of 10:1 in a volume of 200μL. Effector cells were treated with or w/o 10 µg/mL CD16xCD33 BiKE 30 min prior to incubation.

## Results

### Dose-dependent NK cell response to CD16xCD33 BiKE stimulation

**Fig. 1:**
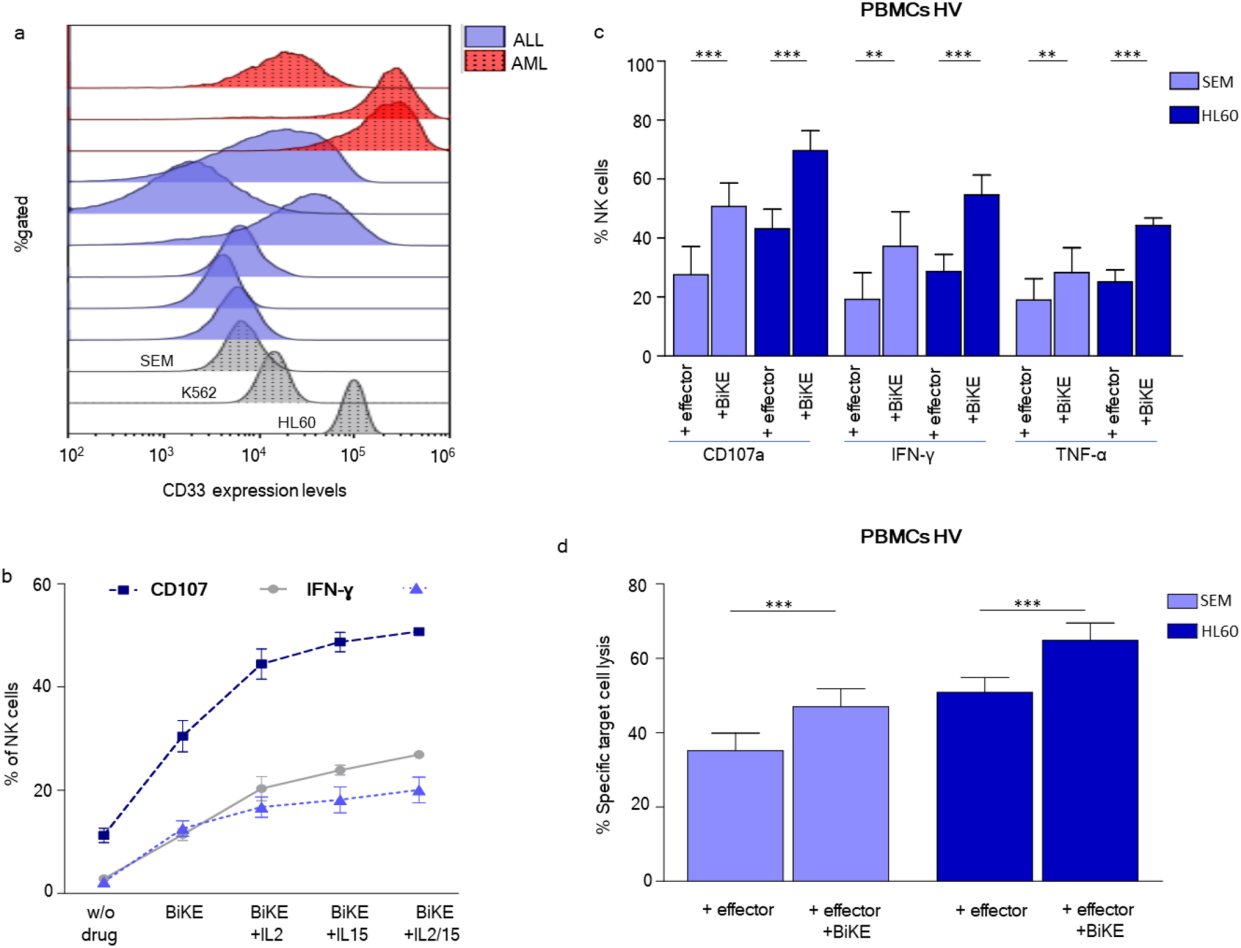
CD16xCD33 BiKE enhances NK cell degranulation and cytokine release against CD33^+^ cell lines. **a** CD33 expression levels of the human acute promyelocytic leukemia cell line HL60/K562 (gray-dotted histogram), the human acute lymphoblastic cell line SEM (gray-dotted histogram), 6 primary ALL patients (light blue histogram) and three primary AML patients (red dotted histogram). **b** PBMCs from healthy donors were coated with 10 μg/mL of the CD16xCD33 BiKE alone or stimulated with IL-2, IL-15 or both of them and cocultured with CD33^+^ SEM targets. **c** Following depletion of CD33^+^ cells, PBMCs were stimulated overnight with IL2, coated with 10 µg/mL CD16xCD33 BiKE and cocultured with HL60 (*n* = 9) and SEM (*n* = 9) targets. NK cell degranulation, intracellular IFN-ɣ, TNF-α production and **d** cytotoxic lysis (PBMCs vs. HL60, *n* = 15, PBMCs vs. SEM, *n* = 10) were measured via flow cytometry. CFSE PI (propidium iodide) double positive cells were measured as cell death. Bars represent the mean expression from 9 healthy individuals **c** and 15/10 healthy individuals vs. HL60/SEM **d**, error bars represent standard error of the mean (SEM), statistical significance was determined by Mann–Whitney U test or paired t test (*p < 0.05, **p < 0.01, ***p < 0.001)

In a first step we determined the sensitivity of the CD16xCD33 BiKE construct, concerning the issue of low expression levels of CD33 on primary biphenotypic childhood ALL blasts. To this end, we tested whether the CD16xCD33 BiKE induces NK cell effector function not only against the CD33^hi^ HL60 cell line but also against the CD33^lo^ SEM cell line, showing a low to very low CD33 expression profile, comparable to CD33^+^ primary ALL blasts (Fig. 1a). A dose-dependent increase in NK cell effector function (CD107a and cytokine production (IFN-γ and TNF-α)) was observed in response to the CD16xCD33 BiKE, with the highest increase at 10 µg/mL (Supplementary Figure S1). Based on these results, we selected a dose of 10 µg/mL for CD16xCD33 BiKE for further experiments. Furthermore, we compared the additional contribution of interleukins to CD16xCD33 BiKE stimulation. By adding equivalent molar concentrations (250U) of IL-2, IL-15 or both, CD107a degranulation and cytokine production could be further increased (Fig. 1b).

### CD16xCD33 BiKE enhances allogeneic NK cell effector functions against CD33^hi^ HL60 and CD33^lo^ SEM targets and also against primary ALL and AML targets

**Fig. 2:**
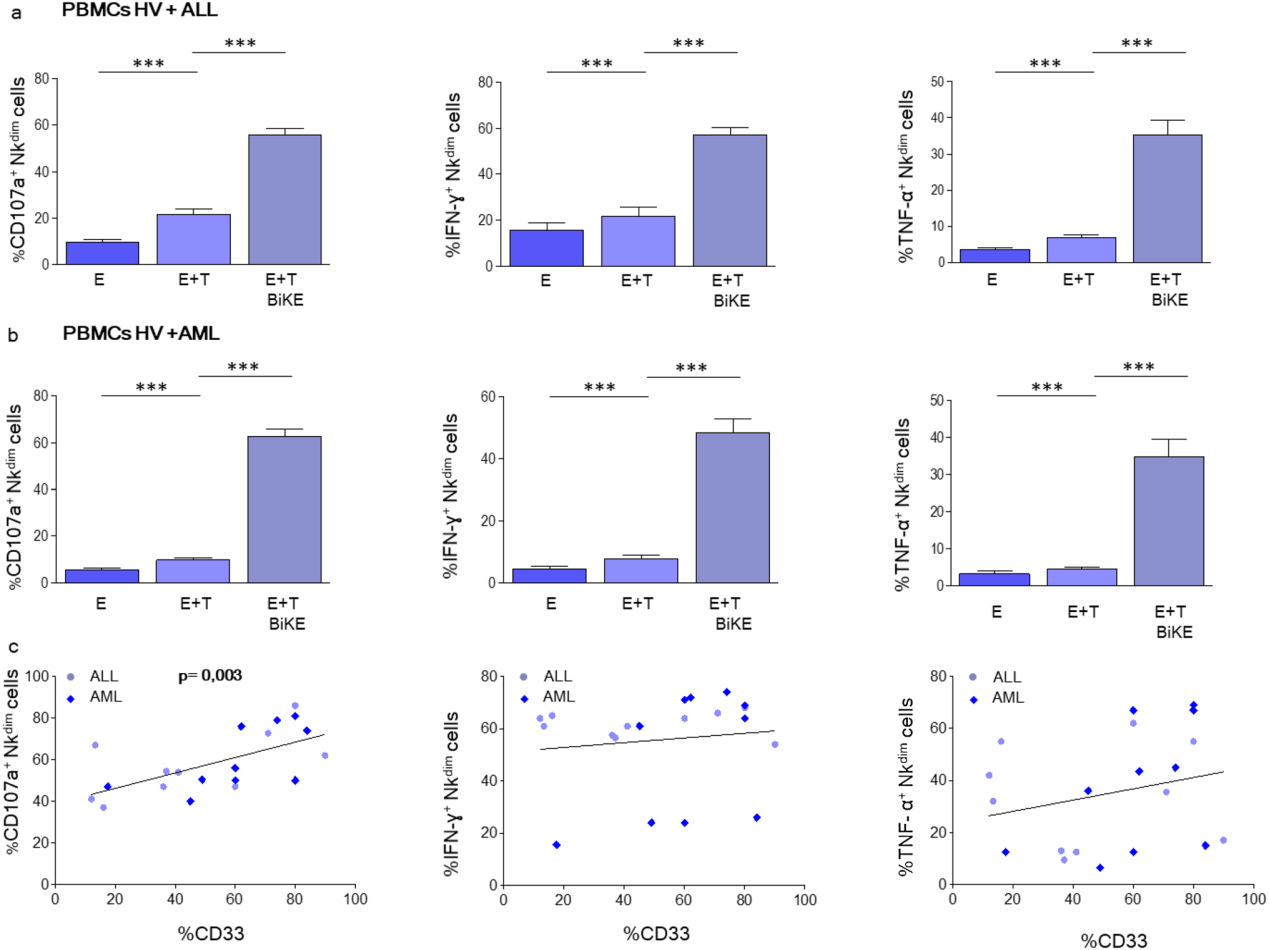
CD16xCD33 BiKE enhances degranulation and cytokine production against pediatric ALL and AML. PBMCs from healthy donors were coated with or w/o 10 μg/mL of CD16xCD33 and cocultured with either pediatric ALL **a** or AML **b** mononuclear cells. CD107a degranulation and intracellular IFN-γ and TNF-α production **(a + b)** were evaluated via flow cytometry analysis. Bars represent the mean expression of two healthy individuals against 10 pediatric leukemic samples, either ALL **a** or AML **b**. **c** Correlation between CD56^dim^ NK cell activity (degranulation, IFN-γ and TNF-α secretion) and CD33 expression (frequency) from 10 leukemic BiKE-treated ALL (circles) and AML (rhombus) patients. Error bars represent standard error of the mean (SEM). Statistical significance was determined by Mann–Whitney U test or paired t test (*p < 0.05, **p < 0.01, ***p < 0.001)

To evaluate the therapeutic potential of the antibody, peripheral blood mononuclear cells (PBMC) from healthy volunteers were treated with CD16xCD33 and cocultured with CD33^hi^ HL60 and CD33^lo^ SEM cells, respectively. NK cell degranulation, TNF-α and IFN-y production were then evaluated by flow cytometry and NK cell-mediated killing by CFSE cytotoxicity assay. Control conditions without antibody did not increase NK cell degranulation and cytokine production compared to the conditions treated with CD16xCD33. NK cell degranulation and cytokine production were significantly increased by the addition of the CD16xCD33 BiKE in the presence of CD33^hi^ HL60 or CD33^lo^ SEM cells (Fig. 1c, Figure S2a–c). The comparatively low CD33 expression levels of SEM cells were apparently sufficient to stimulate NK cell effector function via bridging with CD16xCD33 BiKE. Similar observations were made regarding NK cell-mediated cytotoxicity: CD16xCD33 BiKE induced target cell death as measured by CFSE assay (Fig. 1d, Figure S2c). Thus, we next compared the effector functions of healthy allogeneic NK cells against primary ALL and AML blasts. Thawed PBMCs were incubated with CD16 × 33 BiKE and cocultured with primary ALL (Fig. 2a, Figure S3a) or AML blasts (Fig. 2b, Figure S3b) for 4 h. BiKE treatment led to a significant increase in mobilization of CD107^+^ cytotoxic granula in NK cells from healthy volunteers against primary ALL and AML blasts, underlining the insufficient antileukemic function of NK cells without BiKE treatment. The same applied for cytokine production of IFN-y and TNF-α, which could be significantly boosted by the addition of CD16xCD33 BiKE. A correlation between the percentage of CD33 on primary blasts and NK effector functions appeared to be highly significant (*p* = 0.003) for NK cell degranulation (CD107a). A correlation between higher CD33 expression level and cytokine production could not be observed (Fig. 2c). These data indicate that CD16xCD33 BiKE induces substantial activation of healthy donor NK cells against CD33-expressing tumor targets.

### Functionally deficient NK cells from ALL/AML patients can be re‑stimulated via CD16xCD33 BiKE depending on granzyme B and perforin levels

**Fig. 3:**
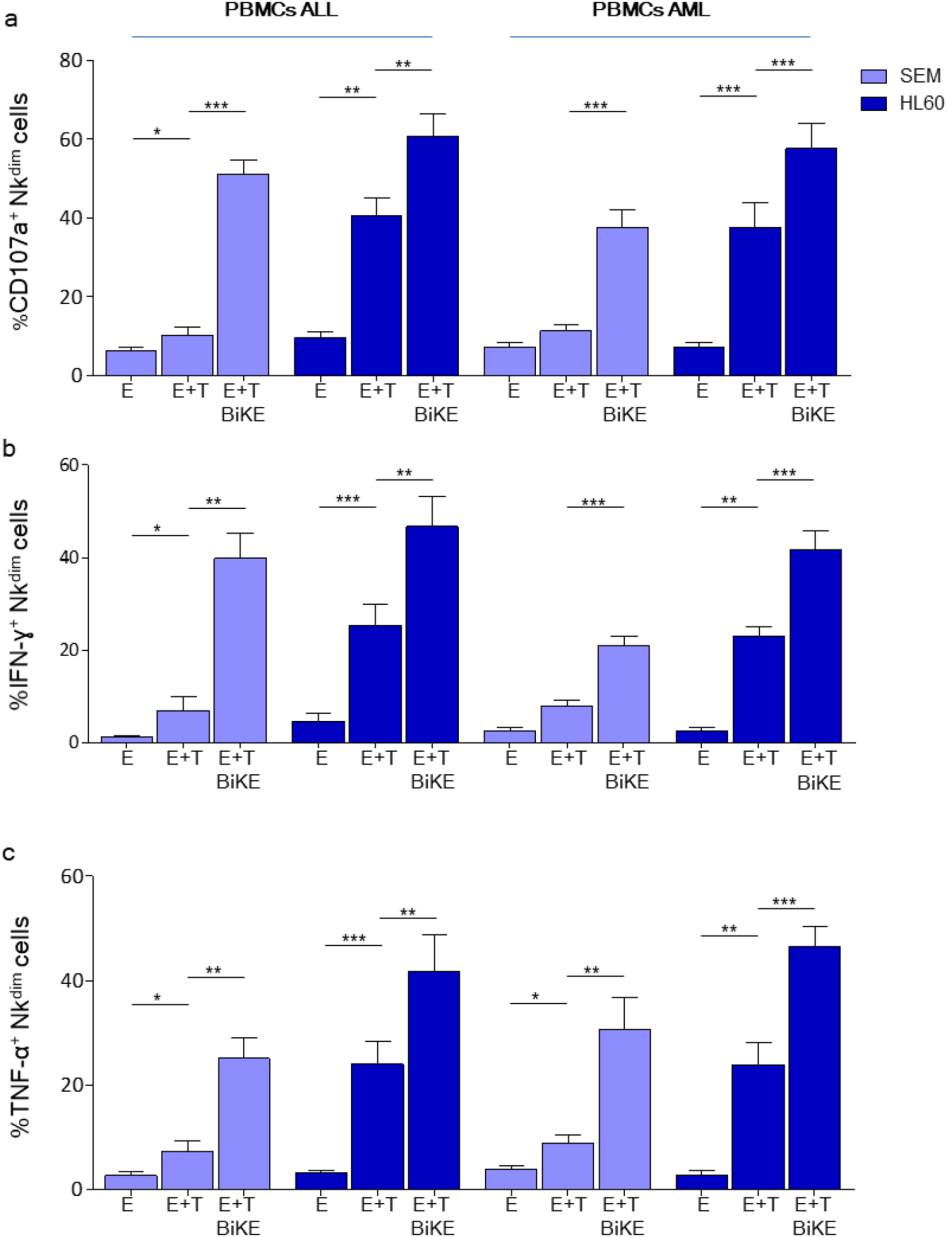
CD16xCD33 BiKE enhances NK cell activity from primary ALL and AML patients against the CD33^+^ target cell lines SEM and HL60. PBMCs from ALL (light blue) and AML (dark blue) patients were coated with or w/o 10 μg/mL of CD16xCD33 and cocultured with SEM and HL60 target cells. **a**–**c** SEM and HL60-induced degranulation and cytokine production of NK cells following BiKE stimulation was measured in six CD33^+^ ALL and AML patients with flow cytometry. Error bars represent standard error of the mean (SEM). Statistical significance was determined by Mann–Whitney U test or paired t test (*p < 0.05, **p < 0.01, ***p < 0.001)

**Fig. 4:**
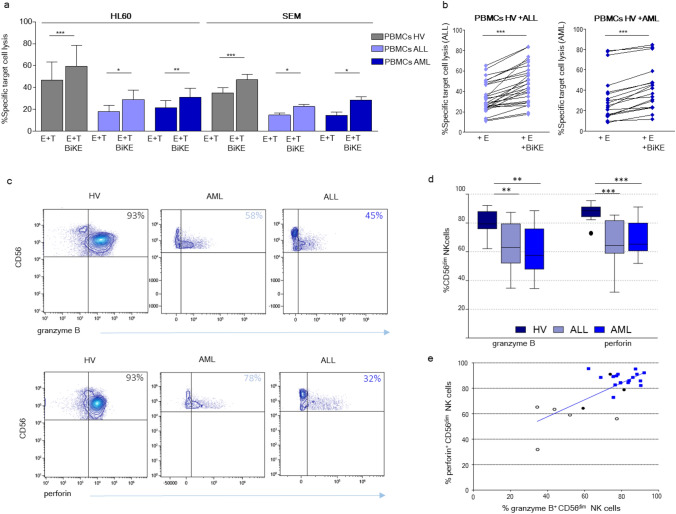
CD16xCD33 BiKE enhances NK cell cytotoxicity against CD33^+^ target cell lines HL60 and SEM in AML and ALL patients: role of granzyme B and perforin. **a** Cytotoxicity of NK cells from healthy individuals (HV; gray, *n* = 19 vs. HL60, 10 vs. SEM), from ALL (filled histograms: light blue, *n* = 5 vs. HL60 and SEM) and AML (filled histograms: dark blue, *n* = 8 vs. HL60 and *n* = 5 vs. SEM) patients against HL60 or SEM cells with or w/o 10 μg/mL of CD16xCD33. Cytolytic lysis was measured via Carboxyfluorescein diacetate succinimidyl ester (CFSE) target labeling. **b** Cytotoxicity of NK cells in three independent experiments from eight healthy individuals against 10 ALL patients (left panel, light blue) and from four healthy individuals against 6 AML patients with or w/o 10 μg/mL of CD16xCD33. **c** Granzyme B and perforin expression levels on CD56^+^ NK cells are shown for a representative AML and ALL patient and a healthy donor. **d** Box plots showing intracellular staining (ex vivo) of granzyme B and perforin in CD56^dim^ NK cells from 17 healthy volunteers (HV; dark blue), 8 AML (light blue) and 8 ALL patients (blue). **e** Correlation between granzyme B and perforin expression in CD56^dim^ NK cells from 17 healthy volunteers (squares) and 8 leukemic BiKE-treated patients (circles). Patients with normal (closed circles) or deficient (open circles) NK cell cytotoxicity (defined as lysis of HL60 cells > 35% or < 35%, respectively) are indicated. Error bars represent standard deviation. Statistical significance was determined by a paired or unpaired t test (*p < 0.1, **p < 0.01, *** *p* < 0.001)

Next, we tested the ability of CD16xCD33 BiKE to increase CD107a mobilization, cytokine production and cytotoxicity in NK cells from leukemic patients against CD33^+^ target cells. Indeed, NK cell degranulation and cytokine production were significantly increased by the addition of the CD16xCD33 BiKE in the presence of CD33^hi^ HL60 (Fig. 3a, b and c, Figure S4a-d). However, killing of target cells by patient NK cells could only be moderately increased via CD16xCD33 BiKE with significant upregulation seen in ALL and also AML patients (Fig. 4a, Figure S5a-b). We next compared killing capacity of healthy allogeneic NK cells against primary ALL and AML blasts. Thawed PBMCs were incubated with CD16 × 33 BiKE and cocultured with primary ALL (Fig. 4b, left panel) or AML blasts (Fig. 4b, right panel) for 4 h. BiKE treatment led to a significant increase in NK cell-mediated cytotoxicity against primary ALL and AML blasts (Fig. 4b). Since NK cells from ALL and AML patients showed normal mobilization of CD107^+^ cytotoxic granula but impaired cytotoxicity, we next explored whether granules were properly armed with the cytotoxic molecules granzyme B and perforin. Intracellular staining of the two molecules revealed a substantially reduced arming of cytotoxic granules in the majority of CD56^dim^ NK cells from ALL and AML patients compared to healthy volunteers (Fig. 4c, d). In most cases, both molecules were reduced simultaneously (Fig. 4e) suggesting a functionally deficient NK cell phenotype. The highest killing efficiency was measured in the two samples which exhibited the highest levels of perforin and granzyme. The data suggest that the decreased cytotoxic NK cell response after stimulation via BiKE could be at least partly explained by reduced levels of granzyme B and perforin in NK cells from leukemic patients.

## Discussion

Despite constant development of novel treatment options and the associated improvement in the prognosis of children with acute leukemia, recurrent and refractory cases in particular still pose a major clinical challenge. The chances of cure are still unsatisfactory, in particular in AML and biphenotypic ALL patients. In this context, the surface antigen CD33, which is stably expressed on leukemic blasts in AML and a subset of pediatric ALL provides a potentially useful tumor target. Recently, a therapeutic bispecific killer cell engager (CD16xCD33 BiKE) has been developed, which provides promising results by linking the tumor antigen CD33 with the ADCC-inducing molecule CD16 [11–13], which is mainly expressed on NK cells. The therapeutic potential of redirecting CD16^+^NK cells toward transformed cells was already established for MDS and adult leukemia patients [12, 14] but so far not explored in pediatric AML and biphenotypic ALL.

The present study demonstrates that the CD16xCD33 BiKE is a specific and sensitive reagent that substantially enhances allogeneic NK cell effector functions against primary pediatric AML and ALL blasts. BiKE treatment significantly induced cytotoxic granule mobilization and the secretion of cytokines (IFN-ɣ, TNF-α) in NK cells from all analyzed donors. Moreover, cell surface mobilization of CD107 and cytokine production could be significantly restored via BiKE in primary NK cells isolated from AML patients. A less significant effect of CD16xCD33 BiKE was seen for NK cells of AML and ALL patients. Subsequent analysis revealed moderately decreased NK cell numbers (data not shown) and more importantly a substantial downregulation of granzyme B and perforin, potentially explaining the impaired cytotoxic response against leukemic targets in the majority of patients. Notably, patients with normal levels of granzyme B and perforin exhibited superior functionality upon CD16xCD33 BiKE stimulation compared to patients with low perforin and granzyme levels. In this regard, we had previously shown in patients with myelodysplastic syndrome that the lack of armed granules is a predictor for poor cytotoxicity [15]. It might thus be advisable to determine the levels of granzyme and perforin in the diagnostic process to identify patients with a high likelihood of NK cell effector deficiency. Single therapy approaches with CD16xCD33 BiKE would then be most promising in patients with functionally competent NK cells. Notably, the likelihood of severe side effects like cytokine release syndrome as observed in novel anti-CD19 targeted immunotherapies such as chimeric antigen receptor T cells and bispecific anti-CD19/CD3 antibodies is negligible following BiKE-mediated NK cell surveillance [16].

In patients with functionally deficient NK cells, a promising approach would be to combine the CD16xCD33 BiKE with adoptive transfer of allogeneic NK cells, which in contrast to the patient's NK cells are functionally not compromised. In general, safety and efficacy of allogeneic infusions of NK cells are well established for immunotherapy of hematological malignancies [17] and are comparatively safe due to the lack of GVHD induction in the allogeneic setting [18–20]. In the setting of NK cell therapy, IL-15 emerges as a key player to support the long-term presence of NK cells in the host, as recently reported for CAR NK cell therapy against ALL [21]. Thus, another promising avenue would be the combination of IL-15-producing NK cells with BiKE. In this regard, IL-15 was also put into play by constructing TriKE molecules combining the BiKE features with IL-15 stimulatory properties [13, 14]. It is however currently unclear, if IL-15 delivery via TriKE is as effective as endogenous overexpression of IL-15 in NK cells. A clinical trial is currently underway investigating the safety and efficacy of CD16xIL-15xCD33 TriKE in adults with high-risk myelodysplastic syndromes, refractory/relapsed acute myeloid leukemia or advanced systemic mastocytosis (www.clinicaltrials.gov; NCT03214666).

In summary, we found that not only pediatric AML, which consistently express CD33 on the cell surface but also CD33^+^ ALL, characterized by inferior prognosis, are potential targets for CD16xCD33 BiKE. Our results encourage the implementation and evaluation of this cost-effective off-the-shelf product in pediatric AML and CD33^+^ ALL patients. Especially in relapses of CD33^+^ ALLs associated with CD19 loss these substances could serve as potential maintenance therapy or “bridging” consolidation chemotherapy before hematopoietic stem cell transplantation.

### Supplementary Information

Below is the link to the electronic supplementary material.

Supplementary file1 (PDF 727 kb)

## References

[1] Linet MS, Ries LA, Smith MA (1999). Cancer surveillance series: recent trends in childhood cancer incidence and mortality in the United States. J Natl Cancer Inst.

[2] Matutes E, Pickl WF, Van't Veer M (2011). Mixed-phenotype acute leukemia: clinical and laboratory features and outcome in 100 patients defined according to the WHO 2008 classification. Blood.

[3] Mejstrikova E, Kalina T, Trka J (2005). Correlation of CD33 with poorer prognosis in childhood ALL implicates a potential of anti-CD33 frontline therapy. Leukemia.

[4] Ruggeri L, Capanni M, Casucci M (1999). Role of natural killer cell alloreactivity in HLA-mismatched hematopoietic stem cell transplantation. Blood.

[5] Ljunggren HG, Malmberg KJ (2007). Prospects for the use of NK cells in immunotherapy of human cancer. Nat Rev Immunol.

[6] Cooper MA, Fehniger TA, Turner SC (2001). Human natural killer cells: a unique innate immunoregulatory role for the CD56(bright) subset. Blood.

[7] Lanier LL (2003). Natural killer cell receptor signaling. Curr Opin Immunol.

[8] Yokoyama WM, Plougastel BF (2003). Immune functions encoded by the natural killer gene complex. Nat Rev Immunol.

[9] Scott AM, Allison JP, Wolchok JD (2012). Monoclonal antibodies in cancer therapy. Cancer Immun.

[10] Lanier LL (2008). Up on the tightrope: natural killer cell activation and inhibition. Nat Immunol.

[11] Wiernik A, Foley B, Zhang B (2013). Targeting natural killer cells to acute myeloid leukemia in vitro with a CD16 x 33 bispecific killer cell engager and ADAM17 inhibition. Clin Cancer Res.

[12] Gleason MK, Ross JA, Warlick ED (2014). CD16xCD33 bispecific killer cell engager (BiKE) activates NK cells against primary MDS and MDSC CD33+ targets. Blood.

[13] Gleason MK, Verneris MR, Todhunter DA (2012). Bispecific and trispecific killer cell engagers directly activate human NK cells through CD16 signaling and induce cytotoxicity and cytokine production. Mol Cancer Ther.

[14] Vallera DA, Felices M, McElmurry R (2016). IL15 trispecific killer engagers (TriKE) make natural killer cells specific to CD33+ targets while also inducing persistence, In vivo expansion and enhanced function. Clin Cancer Res.

[15] Hejazi M, Manser AR, Frobel J (2015). Impaired cytotoxicity associated with defective natural killer cell differentiation in myelodysplastic syndromes. Haematologica.

[16] Felices M, Lenvik TR, Davis ZB (2016). Generation of BiKEs and TriKEs to improve NK cell-mediated targeting of tumor cells. Methods Mol Biol.

[17] Veluchamy JP, Kok N, van der Vliet HJ (2017). The rise of allogeneic natural killer cells as a platform for cancer immunotherapy: recent innovations and future developments. Front Immunol.

[18] Ruggeri L, Capanni M, Urbani E (2002). Effectiveness of donor natural killer cell alloreactivity in mismatched hematopoietic transplants. Sci.

[19] Miller JS, Soignier Y, Panoskaltsis-Mortari A (2005). Successful adoptive transfer and in vivo expansion of human haploidentical NK cells in patients with cancer. Blood.

[20] Curti A, Ruggeri L, D'Addio A (2011). Successful transfer of alloreactive haploidentical KIR ligand-mismatched natural killer cells after infusion in elderly high risk acute myeloid leukemia patients. Blood.

[21] Liu E, Marin D, Banerjee P (2020). Use of CAR-transduced natural killer cells in CD19-positive lymphoid tumors. N Engl J Med.

