# Supplementary material for: CD16xCD33 Bispecific Killer Cell Engager (BiKE) as potential immunotherapeutic in pediatric patients with AML and biphenotypic ALL
Source: Cancer Immunol Immunother. 2021 Aug 16;70(12):3701–8. doi: 10.1007/s00262-021-03008-0 (PMC8571204; doi:10.1007/s00262-021-03008-0)
Supplement: Supplementary file 1 — Supplementary file1 (PDF 727 kb) [file 262_2021_3008_MOESM1_ESM.pdf]

S1

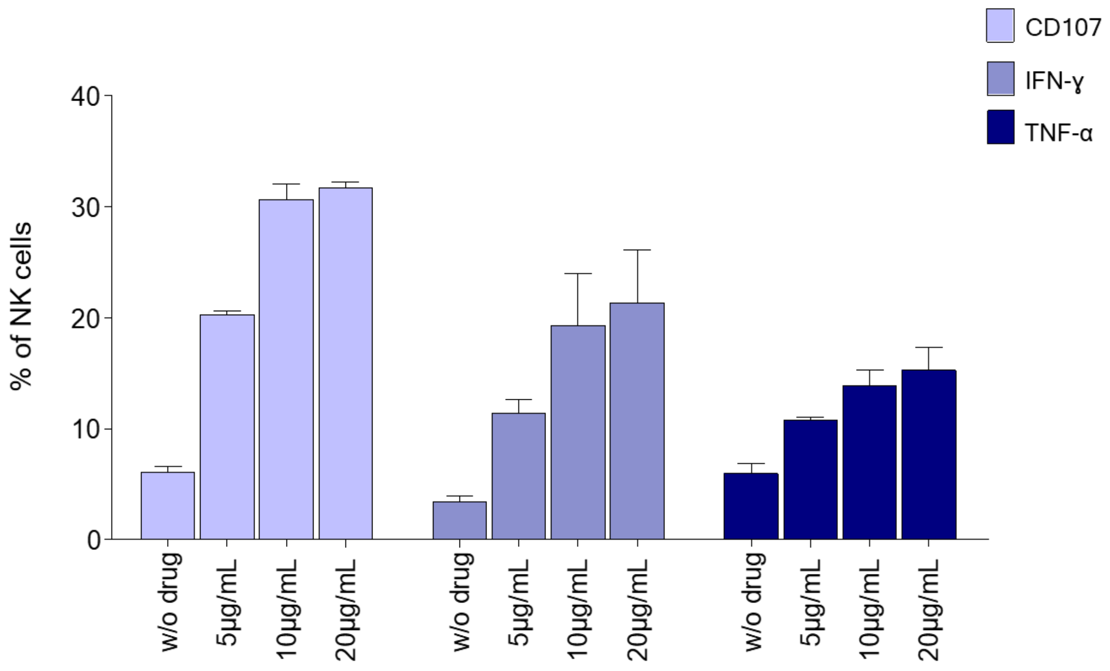

Figure S1: ***CD16xCD33 BiKE enhances NK cell degranulation and cytokine release in a dose-dependent manner against CD33⁺ cell lines.*** PBMCs from healthy donors (n=6) were cocultured with $CD33^+$ SEM targets (E:T ratio 10:1) and treated with the indicated ratios of BiKE. CD107a degranulation and intracellular cytokine production (IFN-ɣ, TNF-α) were measured via flow cytometry.

S2

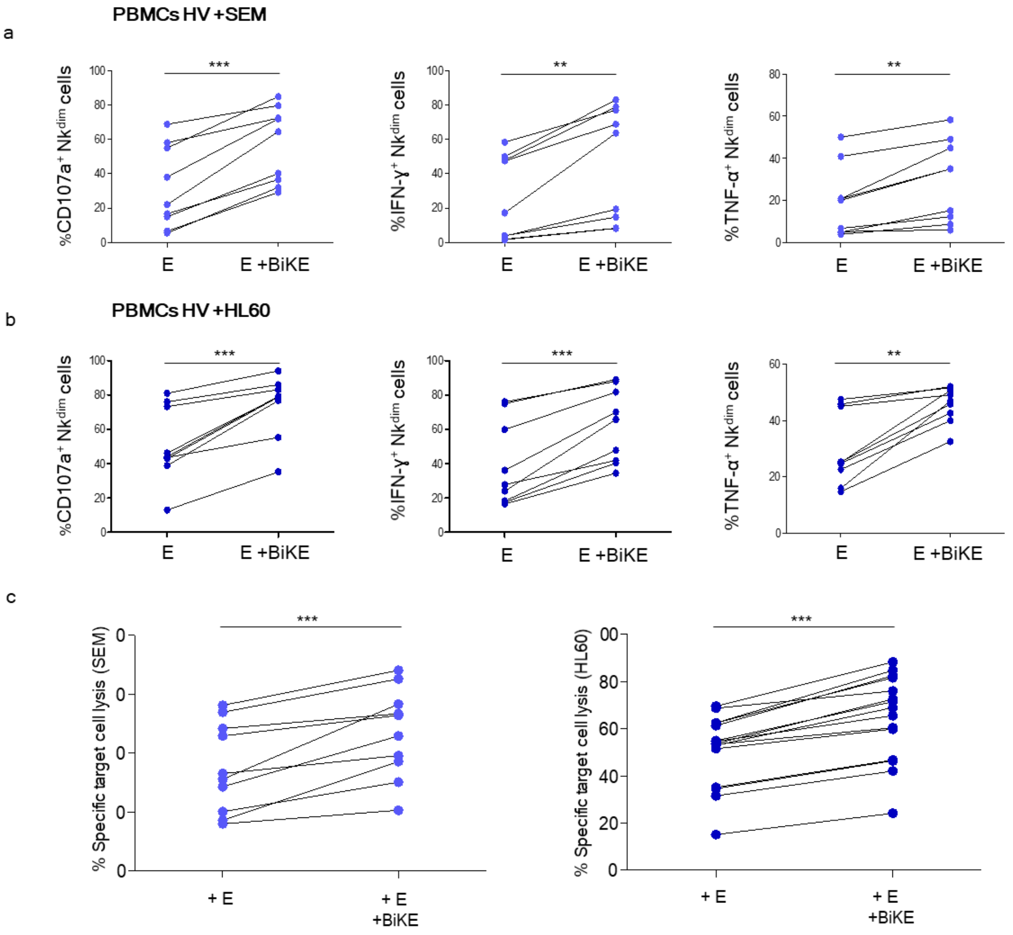

Figure S2: ***CD16xCD33 BiKE enhances NK cell degranulation and cytokine release against $CD33^+$ cell lines.*** Following depletion of CD33+ cells, PBMCs from healthy donors (n=9) were cocultured with CD33+ SEM ***(a)*** or HL60 ***(b)*** targets (E:T ratio 10:1) and treated with (+E +BiKE) or without BiKE (+E) . CD107a degranulation and intracellular cytokine production (IFN-ɣ, TNF-α) were measured via flow cytometry. ***(c)*** Specific target cell lysis was measured via CFSE Assay. Following depletion of CD33+ cells, PBMCs from healthy donors (n=10/15) were cocultured with CD33+ SEM (left panel) or HL60 (right panel) targets (E:T ratio 10:1) and treated with (+E +BiKE) or without BiKE (+E). CFSE PI (propidium iodide) double positive cells were measured as cell death. Each single connected data point labels one individual donor against the respective target, with or without BiKE. Statistical significance was determined by Mann-Whitney U test or paired t-test (*p<0.05, **p<0.01, ***p<0.001).

S3

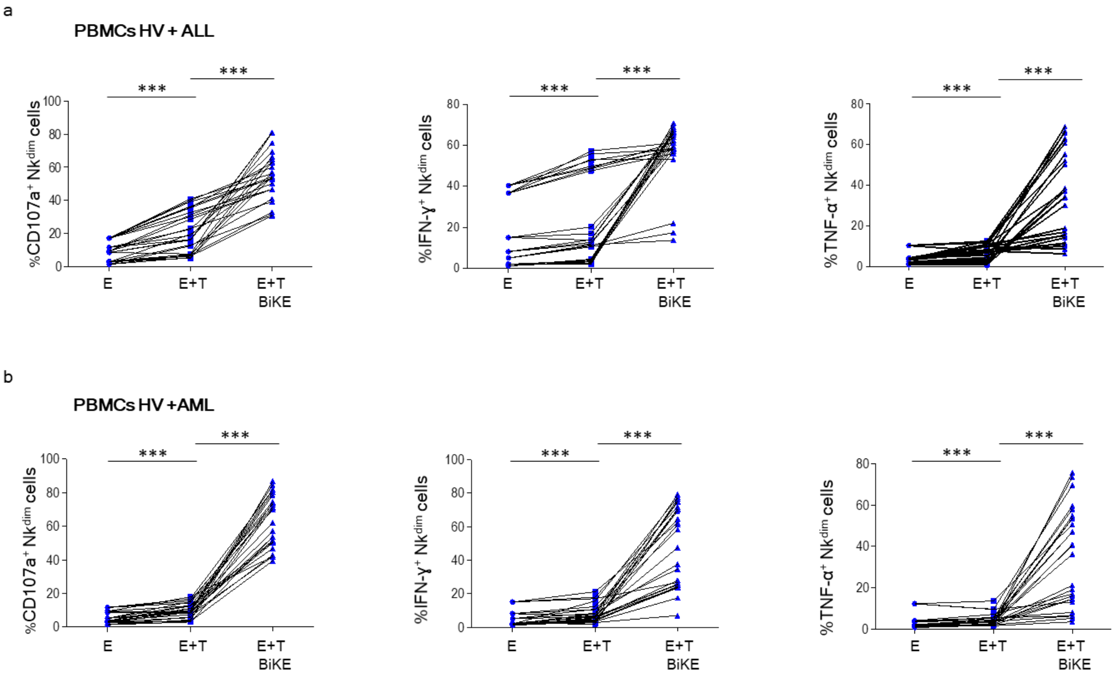

Figure S3: ***CD16xCD33 BiKE enhances degranulation and cytokine production against pediatric ALL and AML.*** PBMCs from healthy donors (n=8) were coated with or w/o 10 µg/mL of CD16xCD33 and cocultured with either pediatric ALL ***(a)*** or AML ***(b)*** mononuclear cells. CD107a degranulation and intracellular IFN-γ and TNF-α production ***(a+b)*** were evaluated via flow cytometry analysis. Bars represent the mean expression of two healthy individuals against 10 paediatric leukemic samples, either ALL ***(a)*** or AML ***(b)***, (E= spontaneous degranulation of effector cells, E+T= effector + target cells without BiKE, E+T BiKE= effector + target cells plus BiKE). Each single connected data point labels one individual donor against the respective target, with or without BiKE. Statistical significance was determined by Mann-Whitney U test or paired t-test (*p<0.05, **p<0.01, ***p<0.001).

S4

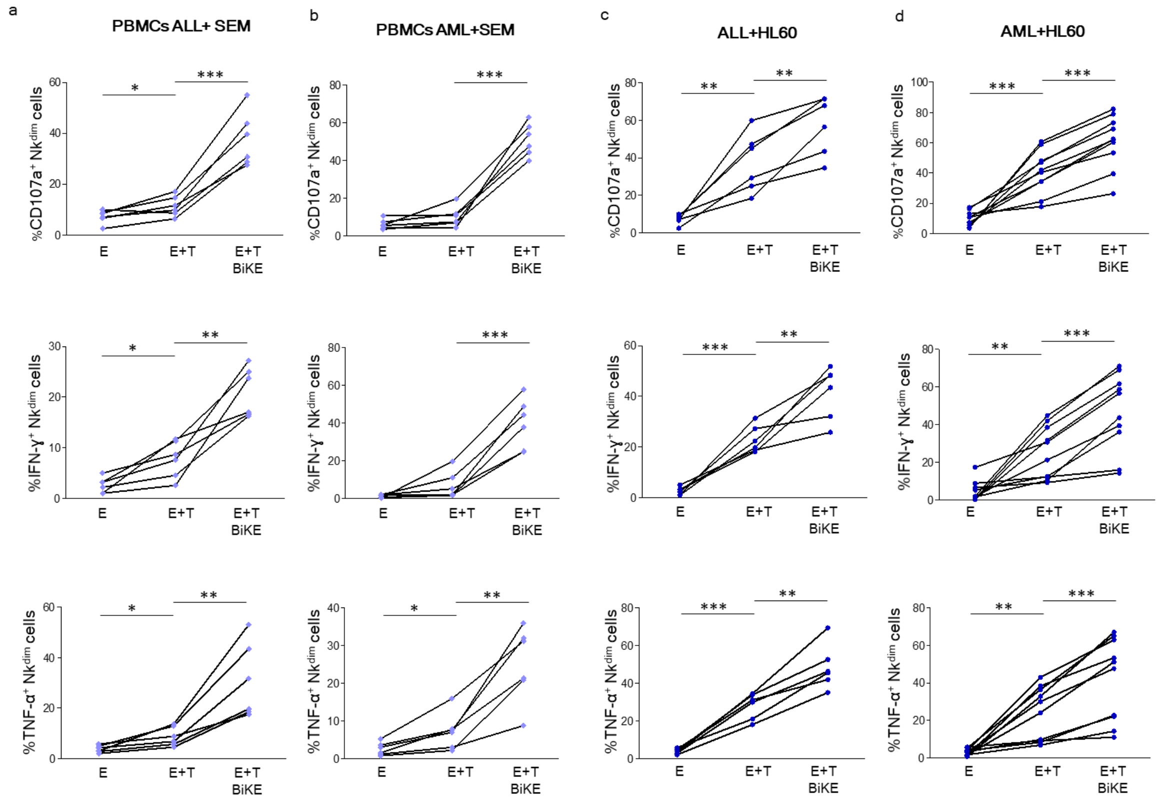

Figure S4: ***CD16xCD33 BiKE enhances degranulation and cytokine production of ALL and AML patients against CD33⁺ cell lines.*** Primary ALL and AML cells (n=6) were coated with or w/o 10 µg/mL of CD16xCD33 and cocultured with either SEM ***(a+b)*** or HL60 ***(c+d)*** cell line. CD107a degranulation and intracellular IFN-γ and TNF-α production ***(a-d)*** were evaluated via flow cytometry analysis. Each single connected data point labels one ALL/AML patient against the respective target, with or without BiKE. Statistical significance was determined by Mann-Whitney U test or paired t-test (*p<0.05, **p<0.01, ***p<0.001).

S5

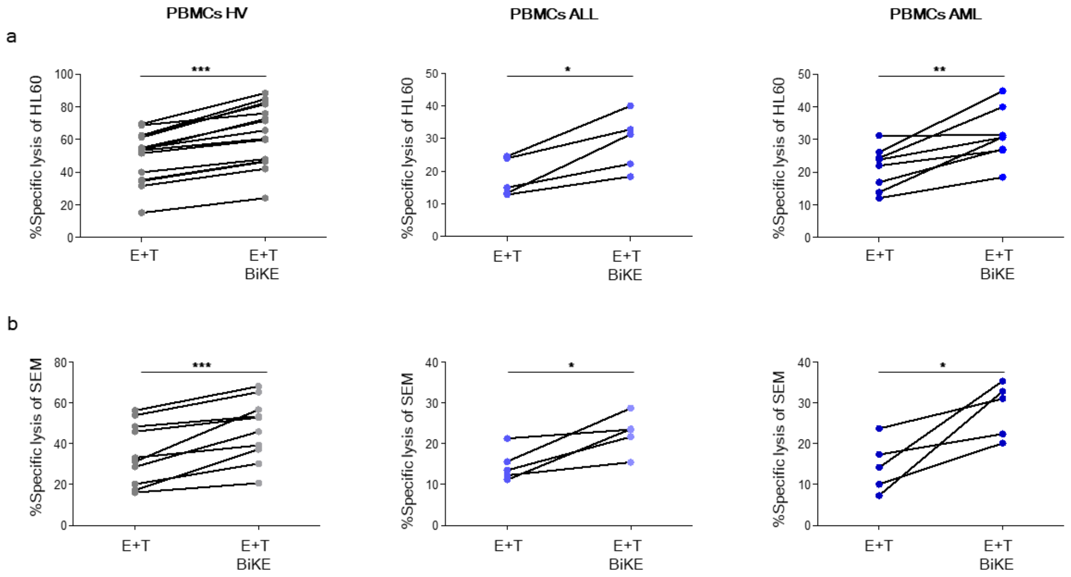

Figure S5: CD16xCD33 BiKE enhances NK cell cytotoxicity of ALL and AML patients against CD33+ cell lines against. Cytotoxicity of NK cells from healthy individuals (HV; grey, n=19 vs. HL60, 10 vs. SEM), from ALL (light blue, n=5 vs. HL60 and SEM) and AML (dark blue, n=8 vs. HL60 and n=5 vs. SEM) patients against HL60 ***(a)*** or SEM ***(b)*** cells with or w/o 10 µg/mL of CD16xCD33. Cytolytic lysis was measured via Carboxyfluorescein diacetate succinimidyl ester (CFSE) target labeling. Each single connected data point labels one HV, ALL or AML patient against the respective target, with or without BiKE. Statistical significance was determined by Mann-Whitney U test or paired t-test (*p<0.05, **p<0.01, ***p<0.001).
